# Resource dilution effect rather than resource concentration hypothesis explains the patterns of pre‐dispersal seed predation of an African cycad along an elevational gradient in South Africa

**DOI:** 10.1002/ece3.70209

**Published:** 2024-08-27

**Authors:** Kantakwa Grégoire Sadiki, Kowiyou Yessoufou, Terence N. Suinyuy

**Affiliations:** ^1^ Department of Geography, Environmental Management and Energy Studies University of Johannesburg Johannesburg South Africa; ^2^ School of Biology and Environmental Sciences, Faculty of Agriculture and Natural Sciences University of Mpumalanga Mbombela South Africa; ^3^ School of Life Sciences University of Kwazulu‐Natal Pietermaritzburg South Africa

**Keywords:** *Encephalartos villosus*, extinction risk, reproduction failure, seed predation

## Abstract

The genus *Encephalartos* is entirely endemic to Africa, and like most cycad species, the genus is at risk of extinction. One of the threats jeopardising the future of the genus is reproduction failure, a failure that is still poorly understood. Our objective was to investigate what predisposes *Encephalartos* species to seed damages through predation, a potential cause of reproduction failure. We collected functional traits of 430 individuals of *Encephalartos villosus*, as well as data on pre‐dispersal seed predation, habitat type and elevation in the Origi Gorge Nature Reserve, South Africa. Then, we analysed our data by fitting a structural equation model. We found that plants tend to be taller when moving from open to close habitat, whereas plant height tends to increase along elevation. In addition, taller plants tend to have more leaves, and plant canopy size shows significant positive relationship with elevation, plant height and number of leaves. These findings suggest a leaf height–canopy dimension strategy perhaps in response to environmental stresses imposed by elevation. We tested the effects of habitat types on seed production. Although there were significantly more seeds in open habitats, open habitats showed the lowest proportion of predated seeds. Finally, we tested the effects of elevation on seed production. We found that seed production decreases along elevation while the proportion of predated seeds increases. Under the resource concentration hypothesis, these findings (where there are more resources, predation is low) are unexpected, suggesting rather that it is the resource dilution effect that matches the pre‐dispersal seed predation patterns in our study area. We suggest that anthropogenic pressures at lower elevation due to easy access may cause seed predators to shift towards higher elevation where they cause heavier damage to seed, thus perhaps contributing to the extinction risk of the genus Encephalartos.

## Graphical abstract

Encephalartos species are at risk of extinction, and seed loss through pre‐dispersal predation is one of the threats to the survival of these species. We found here that open habitat and plant functional traits predispose *Encephalartos villosus* – used as a model species – to pre‐dispersal seed predation. We recommend to avoid any anthropogenic disturbances that may open forest canopy if we are to limit seed predation of this species.

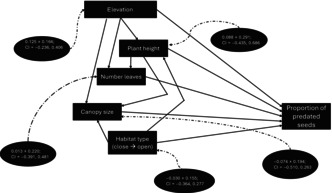

## 1 INTRODUCTION

The resource concentration hypothesis predicts that the population density of specialist herbivorous insects is high where plant‐based resources (e.g. leaves, seeds, etc.) are abundant (Kèry et al., 2001; Ostergard & Ehrlén, 2005; Root, 1973; Sholes, 2008; but see Grez & Gonzàlez, 1995). Factors shaping this spatial pattern (i.e. intense herbivory where resources are abundant) may include food requirements and diet breadth, dispersal biases, sensory biases (visual vs. olfactory), competitive exclusion and predation (Kunin, 1999). Under the resource concentration hypothesis, the interactions of herbivorous insects with plants were suggested as an ecological force regulating the populations of both plants and insects (Heard & Remer, 2008; Myers et al., 1981).

To test this hypothesis along an elevational gradient, one needs to first ask: where are resources concentrated (low, mid‐ or high elevation)? Different patterns of resources along elevation have been reported, depending on how resources were approximated (biomass, seeds, leaves, etc.). For example, a recent study revealed that resources (plant biomass) increase with elevation but not indefinitely, that is, following an unimodal relationship (Lee et al., 2021; see also Thakur et al., 2024), implying that highest concentration of resources was found at mid‐elevation. Olejniczak et al. (2018) also demonstrated an elevation dependence of seed production but revealed that this dependence is species specific. This species specificity imposes to investigate the spatial patterns of resource concentration as a first step if we are to test the hypothesis. Once this spatial pattern is determined, we would expect, as a consequence of the resource concentration hypothesis, that the intensity of herbivory would be high where resources are mostly located (see also Stephens & Myers, 2012). We tested this hypothesis on an African cycad species in South Africa.

Cycads have been considered ‘living fossils’ for a long time because of their long‐term morphological stasis. Globally, over 360 extant cycad species in 10 genera are documented (Govaerts et al., 2021; Yessoufou et al., 2017), of which the genus *Encephalartos* with its 65 species is entirely endemic to Africa (Calonje et al., 2023; Cousins & Witkowski, 2017). *Encephalartos* spp. are dioecious, perennial plants, occurring in forests, grasslands and savannas (Donaldson, 2008). Forest and grassland species hardly tolerate other ecosystems while savanna species are ecologically more tolerant, occurring in open and closed habitats (Donaldson, 2008). In terms of lifeforms, there are not only species with subterranean stems but also dwarf species and trees. *Encephalartos* spp. are long‐lived species with a lifespan ranging from ~150 years (e.g. *E. villosus*) to >1000 years (e.g. *E. cycadifolius*; Raimondo & Donaldson, 2003).

Unfortunately, over 68% of extant cycad species are threatened with high risk of extinction (IUCN, 2022; Yessoufou et al., 2017), concomitantly putting at risk the unique evolutionary history of cycads (Yessoufou et al., 2017). Although competition with angiosperms is primarily the cause of a limited diversification of cycads (Lupia et al., 1999), the extant cycad faces tremendous threats mostly linked to anthropogenic pressures (Mankga & Yessoufou, 2017). Specifically, Mankga and Yessoufou (2017) identified nine major threats jeopardising the future of cycads, including, in order of importance, habitat loss, overcollection, fire, reproduction failure, deforestation, medicinal usage, grazing, flood/drought and invasive species. The mechanism driving the reproduction failure in particular is not only poorly understood, but it is reported most frequently in the African *Encephalartos* spp. (Donaldson, 2009; Mankga & Yessoufou, 2017). Reproduction in *Encephalartos* spp. is infrequent and irregular (Donaldson, 2008). Here, we argue that, by damaging or destroying the seeds, the pre‐dispersal seed predations contribute to reproduction failure in the genus *Encephalartos*. Seed predation by weevils (genus *Antliarhinus*) is common in *Encephalartos* spp. and can destroy up to 90% of their seeds (Donaldson, 1993a, 1993b). The weevil *Antliarhinus zamiae* is the major pre‐dispersal predator of *Encephalartos* seeds in Southern Africa, where it has been detected on 14 *Encephalartos* species (Donaldson, 1991).

Then, how do the seeds of the African cycad *Encephalartos* are damaged or predated? The weevil *Antliarhinus zamiae* grows within cycad seeds (Donaldson, 1991), especially in female cones (Suinyuy et al., 2010). *Antliarhinus* spp. prey heavily on cycad seeds and ovules, and their larvae develop and feed exclusively in it (Donaldson, 1993a; Oberprieler, 1995). Donaldson (1992) reported that the female *Antliarhinus* drills a hole in the ovule and inserts the ovipositor to lay eggs in it. As a result, this seed predation may cause up to 90%–100% seed loss (Donaldson, 1993a; Suinyuy et al., 2010). This rate of seed loss due to the weevil's feeding behaviour is a concern regarding the future of *Encephalartos* spp. (Donaldson, 1992, 1993a; Giddy, 1974; but see Raimondo & Donaldson, 2003). This seed predation in *Encephalartos* causes reproduction failure and poor recruitment, which is a potential conservation problem that significantly heightens the extinction risk of cycad species (Mankga & Yessoufou, 2017). Amsberry and Maron (2006) clarified that insect herbivores have strong negative effects on plant fertility. In response to heavy seed loss, *Encephalartos* spp. are also known to mast (Donaldson, 1993b), and mast seeding promotes regeneration and the conservation of rare species (Pearse et al., 2021).

In such context, a follow‐up question of interest is as follows: What promotes or predisposes cycad seeds to pre‐dispersal predation by the weevil *Antliarhinus zamiae*? We argue that environmental variables as well as several functional traits predispose *Encephalartos* species to seed predation (Kolb et al., 2007). Such environmental and functional traits may include elevation, plant canopy size, plant height, habitat types and number of leaves (Daco et al., 2021; Kolb et al., 2007) on the following ground. Regarding the potential effect of elevation, we assume that the pre‐dispersal seed predation would decrease along elevation (O'Dowd & Gill, 1984; Wright, 1990), and this may be the consequence of a reduction in insect population along elevation (Jump et al., 2009; Zhao et al., 2023). Several studies reported that cycad seed predators tend to be rare in high altitudes than in lowlands (e.g. Suinyuy & Johnson, 2018), making elevation a geographic barrier to seed predation. A decrease in the proportion of damaged seeds at high elevation is likely the result of the negative effects of low temperature on larval development at that elevation (Lee & Kotanen, 2015). We also expect large plant canopy to be conducive to seed predation (Kolb et al., 2007; Leimu et al., 2002). Findings suggest that larger canopy predisposes to high proportion of damaged seeds (Kolb et al., 2007). In their study, Kolb et al. suggested that high seed predation where canopy size is large may simply mirror the habitat preference of the moth which might prefer to oviposit in close habitats, a habitat type that may ensure safety for the eggs and juvenile of seed predators than the open habitats (Leimu et al., 2002). Since large canopy implies more leaves, we expect that large canopy would lead to herbivore abundance and increase seed predation (Li et al., 2023). This expectation is grounded in the resource concentration hypothesis which predicts that insect herbivores would be more abundant where resources (leaves, seeds, etc.) are abundant (Root, 1973).

How about plant height? Plants found in high elevation are expected to be shorter, exhibiting small canopy as opposed to plants at low elevation. This is because, as one moves to higher elevation, temperature decreases and low temperature is a limiting factor to plant growth (Daco et al., 2021; De Frenne et al., 2013; Körner, 2003). This limited growth (height and canopy) at high elevation would be less favourable for a high density or accumulation of seed predators. Also, species in open forests are expected to have a large canopy (Li et al., 2023) and invest more in height (Du et al., 2021; Peters et al., 2019) due to access to solar energy. In addition, we expect the proportion of seed predation to correlate with habitat types, for example, close versus open habitats. Our field observations show that habitats are open at low elevation where we expect to have more seed predators, suggesting that we might find a high number of damaged or predated seeds in open forests at low elevation due to the abundance of insect herbivores promoted by solar energy availability (Li et al., 2023; Suinyuy et al., 2010). Finally, the number of leaves is expected to correlate positively with seed production. For example, Akiyama and Ågren (2012) demonstrated experimentally a reduction in seed production by 22%–60% when 50% of leaves are lost, although this is contingent upon the growing seasons.

In the present study, our aim was to understand what might predispose cycad seeds to damage/predation, using *Encephalartos villosus* as the study model. Specifically, we set three objectives. Our first objective was to understand the intraspecific survival strategy of *Encephalartos villosus* along elevation? Then, we investigated where resources for herbivores (leaves and seeds) are concentrated along the elevational gradient. Finally, we explored whether resource concentration hypothesis explains pre‐dispersal seed predation. To this end, we integrated all the hypotheses we presented in the Introduction about potential links among different functional traits along elevation into one meta‐model (structural equation model; SEM) to meet the three objectives of the study.

## 2 MATERIALS AND METHODS

### 2.1 Study area

**FIGURE 1:**
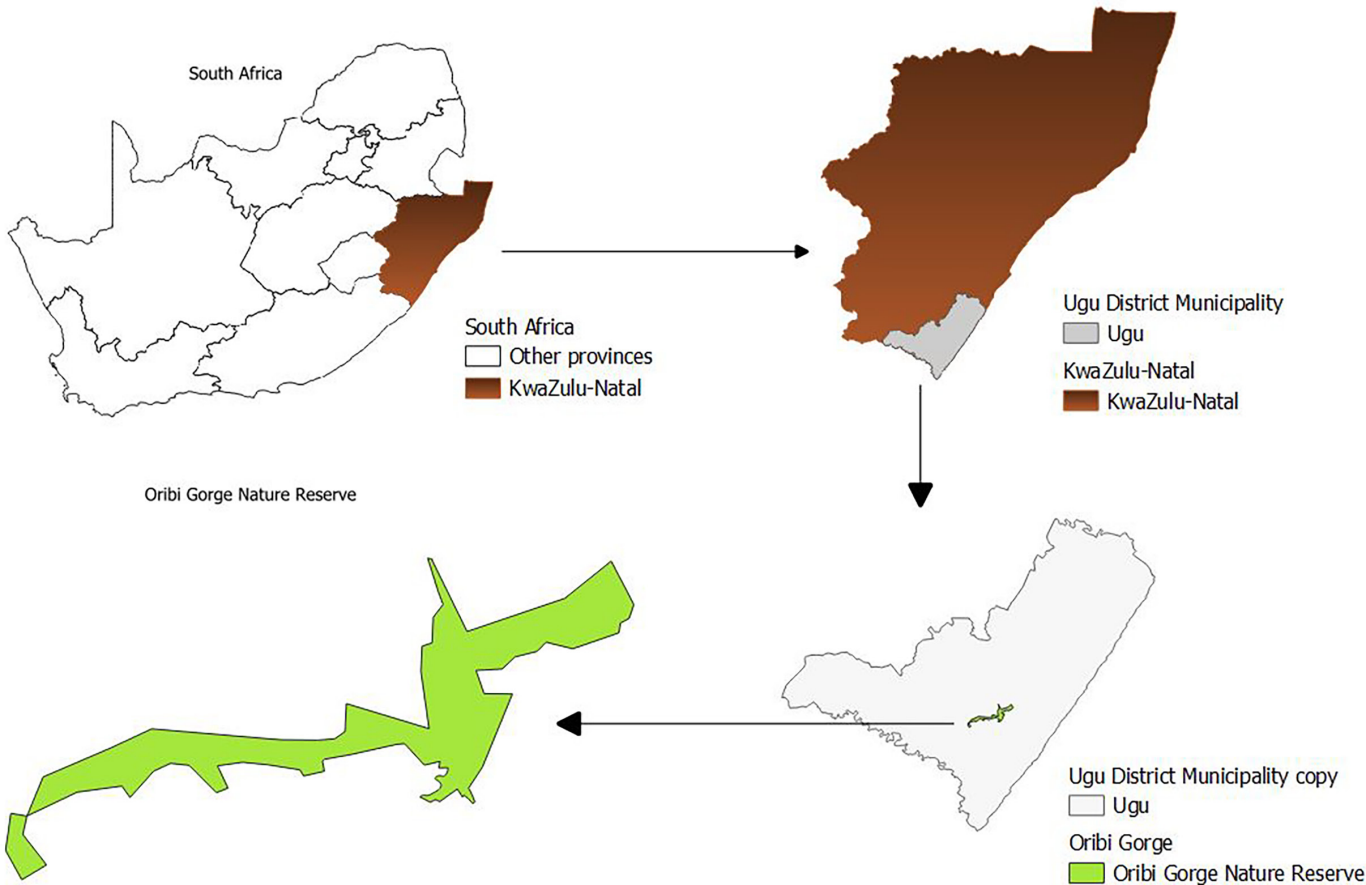
Geographic location of Oribi Gorge Nature Reserve within Ugu District Municipality in the Kwazulu‐Natal Province, South Africa.

This study took place in the Oribi Gorge Nature Reserve (henceforth ‘the reserve’) in South Africa (coordinates are not included due to cycad conservation concerns). The reserve is surrounded by various land‐use types such as sugarcane plantations, wattle and cattle farming. Geographically, the reserve is located within the *Ugu* District Municipality in the South African Province of KwaZulu Natal (Figure 1) (Ezemvelo KZN Wildlife, 2009). Covering the equivalent of a flat surface area of roughly 2000 ha, the reserve is 21 km west of Port Shepstone (Ezemvelo KZN Wildlife, 2009). Within this reserve, elevation ranges between 120 and 680 m asl (Glen, 1972). Furthermore, the reserve is laid on the Msikaba Sandstone Formation which has a marine origin. One characteristic of the reserve is the presence of faults which occurred in the Middle Cretaceous (Glen, 1972). The climatic conditions are characterised by 570–1625 mm of average annual rainfall, with the maximum occurring in summer (October–March) and the lowest in July (Ezemvelo KZN Wildlife, 2009). The local climate is also characterised by an average maximum daily temperature varying between 13 and 23°C within a year (Ezemvelo KZN Wildlife, 2009).

**FIGURE 2:**
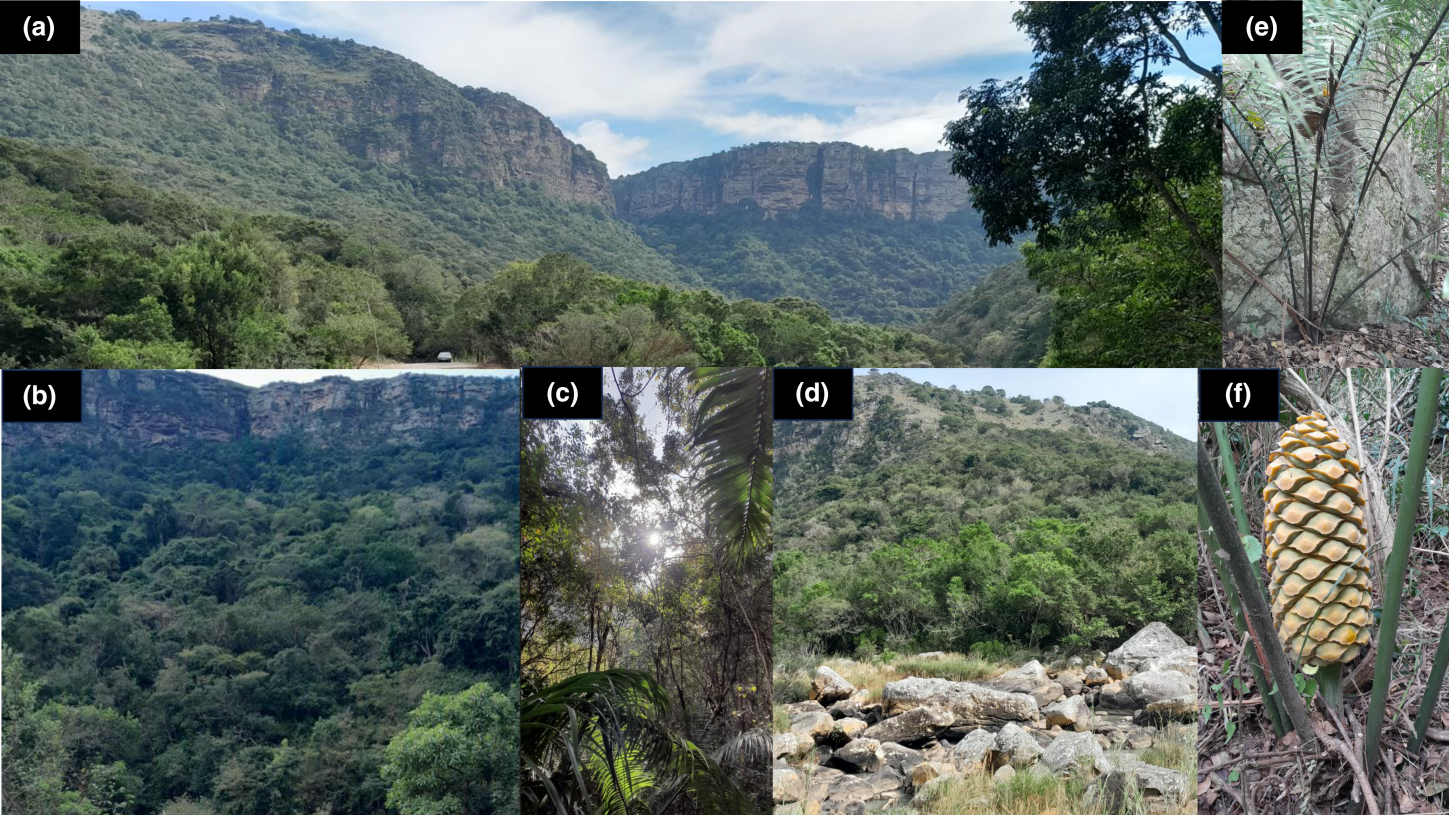
Vegetation types in Oribi Gorge Nature Reserve. (a, b) Forest; (c) a dry Ngongoni Veld; (d) Eastern Valley Bushveld; (e) *Encephalartos villosus*; and (f) female cone of *E. villosus*.

Interestingly, the reserve is species rich with at least 500 documented plant species in different biome types, ranging from evergreen forest (forest‐filled gorge; Figure 2a,b) to grasslands, including a Pondoland Scarp Forest (928 ha). The Pondoland Scarp Forest acts as an Afrotemperate refugia overlapping Afrotemperate and coastal forests (MacDevette et al., 1989; Mittermeier et al., 2004; Van Wyk, 1990), making it a particularly species‐rich biome (Mucina & Rutherford, 2005). The vegetation diversity in the reserve also includes a South Coast Bushland (567 ha), which is an endangered vegetation type, a Pondoland–Ugu Sandstone Coastal Sourveld (287 ha), which with its savanna/grassland structure, is the most threatened vegetation in the reserve. There is also a dry Ngongoni Veld (73 ha; Figure 2c) which is similar to Pondoland–Ugu Sandstone Coastal Sourveld but with a lower species diversity. Finally, within the reserve, there is an Eastern Valley Bushveld (26 ha) which is a savannah biome (Figure 2d) dominated by *Euphorbia* and *Aloe* species (Ezemvelo KZN Wildlife, 2009). These biomes within the reserve are safe haven for threatened, rare, and endemic species, including the cycad genus *Encephalartos* (e.g. *E. villosus*, Figure 2e,f).

### 2.2 Data collection

In 2023, we surveyed all the vegetation types in Oribi Gorge Nature Reserve. During the survey, 430 individuals of *E. villosus* across all life stages were identified. From each individual, we collected data on seven variables including elevation, plant height, plant canopy size, number of leaves, habitat type and seed status (damaged or not by weevils) when cone is present. To measure canopy size, two distances were taken following the two directions North–South and East–West. Each distance is the distance separating the two furthest leaves in both directions. Then, canopy size was calculated as the average of the two distances. In addition, the measurement of the canopy was only done for individuals having at least four leaves. Habitat type was defined as a binary variable: open habitat when the forest canopy is open versus closed habitat when otherwise. Lastly, seed status was determined as damaged seeds by the weevils versus undamaged seeds, and the proportion of damaged seeds was calculated. All data collected are presented in Data S1.

### 2.3 Data analysis

All analyses were performed in R version 4.2.3 (R Core Team, 2021), and the R scripts are provided in Data S2. Prior to analysis, the variables were standardised, and the standardised variables were then analysed.

The variable standardisation was done as follows: *y* = (*x* − $\bar{x}$)/*σ*, where *y* represents the standardised (re‐scaled) observation, *x* represents the unstandardised observation, $\bar{x}$ represents the sample mean and σ denotes the standard deviation of the sample.

In nature, ecosystem function and even the ecology and physiology of plants are determined not by a single factor but by a multiplicity of factors that act synergistically. As such, individual regression analyses cannot capture such network of interactions. However, structural equation models are designed specifically to study a network of interactive factors (Lefcheck, 2016). All the expected relationships between different variables (presented in the Introduction) were simultaneously tested by fitting a structural equation model (SEM) as implemented in the R library *piecewiseSEM* (Lefcheck, 2016). The SEM is made up of four GLM models (see R script): one beta regression to model the *proportion of seeds predated* by the weevils (our main response variable); one negative binomial GLM to model the *number of leaves* on each plant; and two Gaussian GLM to model *plant height* and plant *canopy size*, respectively. We analysed the four models forming the SEM by simultaneously calculating the direct, indirect, and total effects of each predictor of the five response variables in the list of fitted models in the structured equations. Confidence intervals were calculated by bootstrapping (we bootstrap 1000 times estimates for the models in the SEM) using the R function *bootEff* in the R library *semEff* (Murphy, 2022). Then, we used the bootstrap samples to calculate effects and confidence intervals (R function s*emEff*).

## 3 RESULTS

**FIGURE 3:**
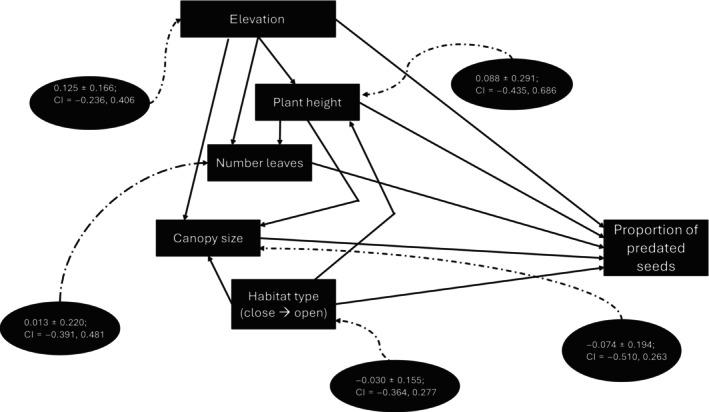
Structural equation model explaining the proportion of pre‐dispersal seed predation of *Encephalartos villosus*. Dashed arrows indicate total effects (direct + indirect; see Table 1) of a given variable, including standard error and confidence interval, on the proportion of predated seeds.

The structure of the metamodel (structural equation model) is presented in Figure 3. The coefficients for all direct and indirect paths are presented in Table 1 and Tables S1–S4.

**TABLE 1:** Coefficients of the model of the proportion of damaged seeds.

Types	Predictors	Effect	Bias	Std. err.	Lower CI	Upper CI
Direct	elevation_s	0.125	−0.016	0.172	−0.244	0.422
	habitat_type_cat.open	−0.020	0.031	0.155	−0.341	0.289
	plant height_s	0.101	0.010	0.163	−0.206	0.433
	number_leaves_ns	0.033	0.019	0.210	−0.364	0.451
	canopy_s	−0.074	0.010	0.194	−0.510	0.263
Indirect	elevation_s	−0.001	0.002	0.036	−0.077	0.075
	habitat_type_cat.open	−0.010	−0.001	0.025	−0.078	0.027
	plant height_s	−0.013	0.018	0.165	−0.343	0.339
	number_leaves_ns	−0.020	0.003	0.054	−0.143	0.077
Total	elevation_s	0.125	−0.014	0.166	−0.236	0.406
	habitat_type_cat.open	−0.030	0.030	0.155	−0.364	0.277
	plant height_s	0.088	0.028	0.291	−0.435	0.686
	number_leaves_ns	0.013	0.022	0.220	−0.391	0.481
	Canopy_s	−0.074	0.010	0.194	−0.510	0.263
Mediators	plant height_s	0.000	0.001	0.026	−0.061	0.054
	number_leaves_ns	0.008	0.015	0.138	−0.255	0.308
	canopy_s	−0.063	0.008	0.167	−0.450	0.228

*Note*: On the variable names, ‘s’ means ‘standardised’ and ‘ns’ means ‘non standardised’.

**FIGURE 4:**
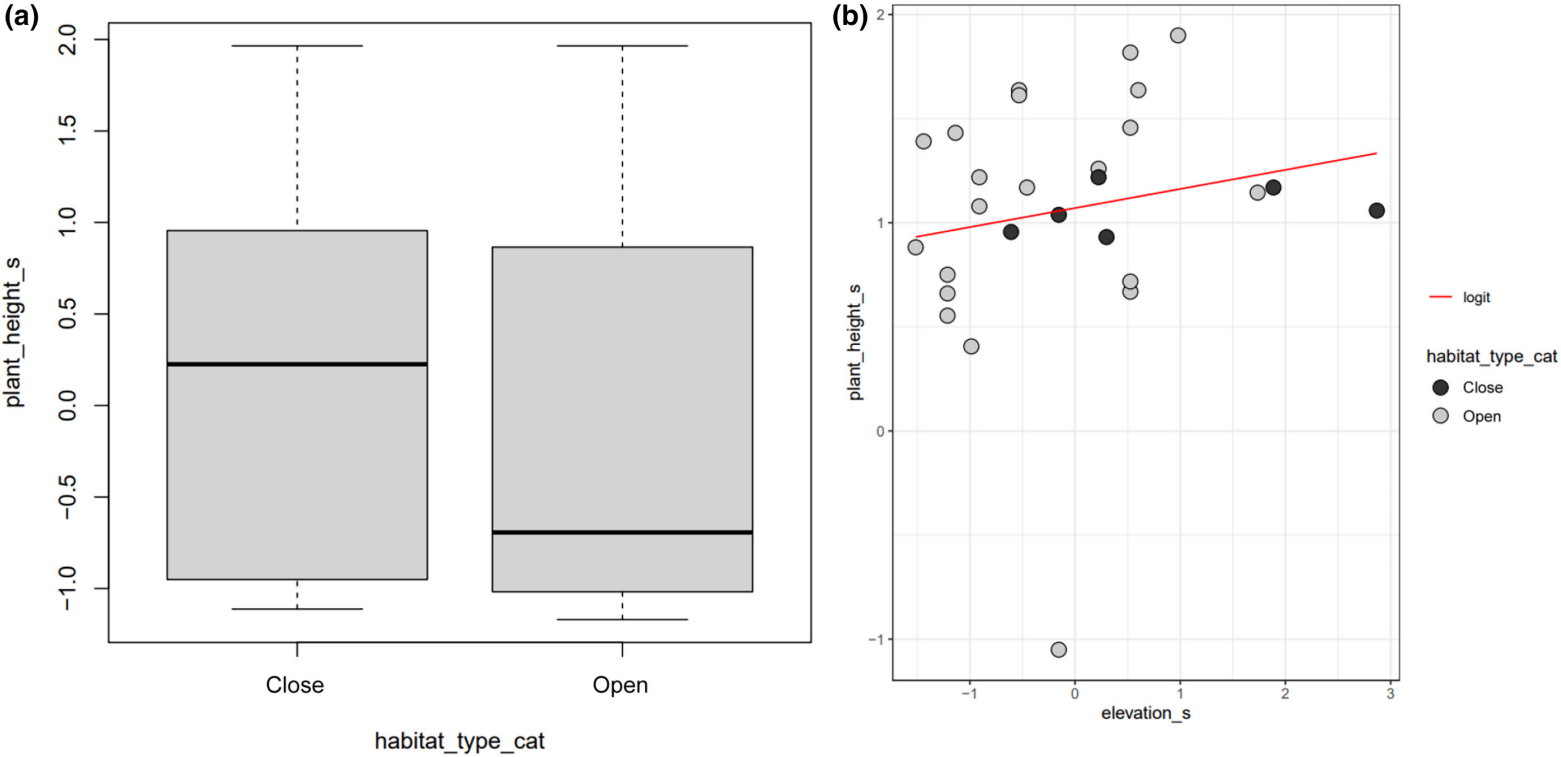
Relationships between plant height and habitat type (a) and elevation (b). Here, height and elevation have been standardised as explained in the methodology.

We found that plants tend to be taller in close habitat and shorter in open habitat (*β* = −0.075 ± 0.047; CI = [−0.172, 0.015]; Figure 4a; Table S1), whereas plant height tends to increase along elevation (*β* = 0.073 ± 0.052; CI = [−0.036, 0.173]; Figure 4b; Table S2). In addition, plant height has a significant positive effect on number of leaves, implying that taller individuals of *E. villosus* tend to have more leaves (total effect in Table S1; *β* = 0.611 ± 0.117; CI = [0.320, 0.776]).

**FIGURE 5:**
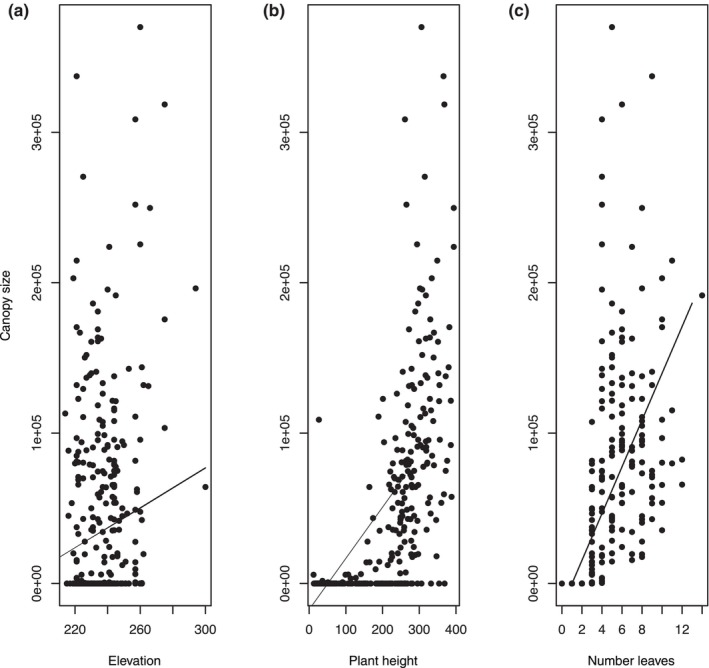
Relationships between canopy size and elevation (a), plant height (b) and number of leaves (c).

Furthermore, plant canopy is significantly influenced by elevation (total effect in Table S3; *β* = 0.120 ± 0.054; CI = [0.017–0.224]; Figure 5a), plant height (*β* = 0.446 ± 0.034; CI = [0.368–0.502]; Figure 5b) and number of leaves (*β* = 0.162 ± 0.041; CI = [0.087–0.237]; Figure 5c).

**FIGURE 6:**
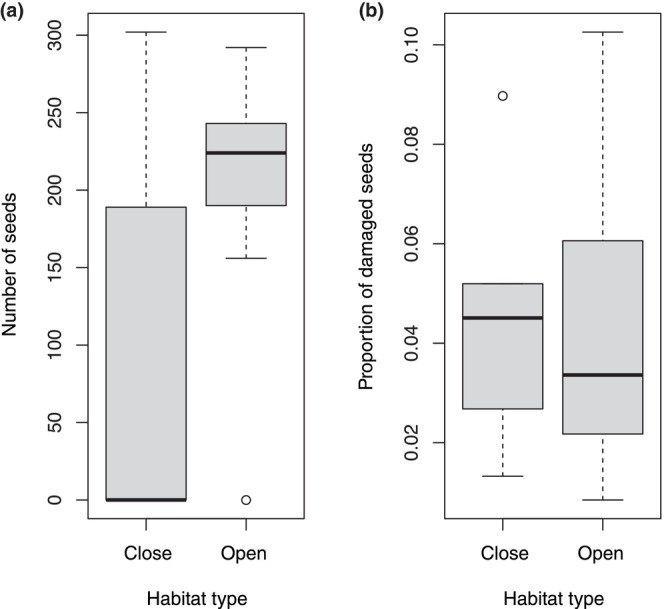
Relationships between habitat type and number of seeds (a) and between habitat type and proportion of damaged seeds (b).

We tested the effects of habitat types on seed production. Although there were significantly more seeds in open habitat (*β* = 0.047 ± 0.046; CI = [0.011, 0.085]; Figure 6a), it is in open habitat that there was the lowest proportion of damaged seeds (*β* = −0.020 ± 0.155, CI = [−0.341, 0.289]; Figure 6b).

**FIGURE 7:**
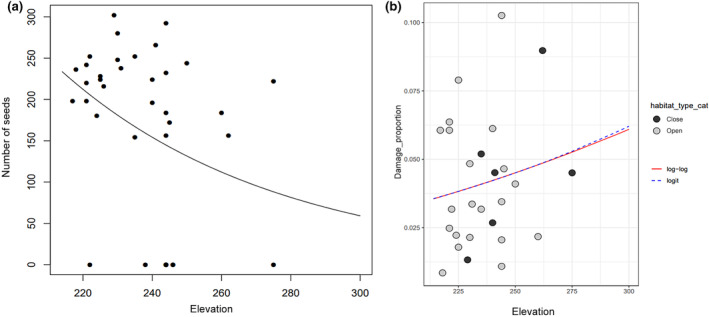
Influence of elevation on seed production (a) and proportion of damaged seeds (b).

Finally, we tested the effects of elevation on seed production. We found that seed production decreases along elevation (Figure 7a; Table S4). Also, among all the variables tested, elevation has the highest effect on the proportion of damaged seeds (total effect; *β* = 0.125 ± 0.166; CI = [−0.236, 0.406]; Figures 3 and 7b), implying that, in terms of proportion, there are more damaged/predated seeds at higher elevation. Also, plant height is the second most influential variable on the proportion of damaged seeds (*β* = 0.088 ± 0.291; CI = [−0.435, 0.686]) such that taller plants tend to bear proportionally more damaged seeds (Table 1).

## 4 DISCUSSION

### 4.1 Intraspecific survival strategy of *Encephalartos villosus* along elevation

We found taller plants in close habitats, whereas shorter plants tend to occupy open habitats. This may be a priori a surprise, given that in open habitats, there is more sunlight available for photosynthesis and growth. However, *Encephalartos villosus* is an understorey species (Donaldson, 1997), implying that it is a shade‐loving plant. It is therefore not surprising that it grows better in its natural ecological niche than not. Also, *E. villosus* in our study area grows under 900–1300 mm annual rainfall (Donaldson, 1997), suggesting that water availability is not an issue.

To further understand the ecology of *E. villosus*, we also investigated the relationships among functional traits. The relationships between ecologically important functional traits are acknowledged as defining an ecological ‘strategy’ dimension (Westoby et al., 2002). Investigating such dimensions help us understand the adaptive measures of plants to their realised niche, that is, why some trait combinations are favoured over others (Westoby et al., 2002; Wright et al., 2007). In the present study, we found that taller plants tend to have more leaves and large canopies while plants with large canopies tend to have more leaves, and elevation selects for plants with large canopy sizes. This clearly indicates that *E. villosus* developed a leaf height–canopy dimension strategy which may reveal physical, physiological or developmental constraints ensuring the adaptation of *E. villosus* to the elevation gradient. This is supported by our finding that elevation tends to select for plants with large canopy sizes. A similar strategy of leaf height–seed dimension strategy was previously proposed between species globally (Westoby, 1998), and the leaf height–canopy dimension strategy we are reporting here for *E. villosus* should be regarded as an intraspecific strategy.

### 4.2 Where are resources (leaves and seeds) concentrated for herbivores along an elevational gradient?

The spatial distribution of the abundance of resources is different depending on the type of resources (e.g. leaves and seeds). For example, we found that plant height increases along elevation, and taller trees tend to have more leaves, suggesting that individuals of *E. villosus* at higher elevations bear more leaves, that is, more food for herbivores. However, the number of seeds decreases along elevation, meaning seed production is high at low elevation. What is the potential consequence of this on seed predation? Since the weevil *Antliarhinus zamiae* predates seeds, and seeds are more abundant at low elevation (see also Jump et al., 2009; Zhao et al., 2023), we expected, under the resource concentration hypothesis (Root, 1973; Tahvanainien & Root, 1972), more weevils and thus more seed predations at low elevation (O'Dowd & Gill, 1984; Wright, 1990). Our prediction was supported in recent studies that reported that cycad seed predators tend to be rare in high altitudes (e.g. Suinyuy & Johnson, 2018), making elevation a geographic barrier to cycad seed predation. In a very specific way, a recent study quantified the relationships between seed predation and elevation and showed that seed predation increased by 17% from 4000 m asl to sea level at a rate of 0.4% increase in seed predation every time one moves 100 m downwards (Hargreaves et al., 2019). This trend may be climatically mediated since an earlier study found a significant relationship between mean annual temperature and actual evapotranspiration (an indicator of food availability) versus seed predation by invertebrates at global scale (Peco et al., 2014).

### 4.3 Does resource concentration hypothesis explain pre‐dispersal seed predation?

Contrary to the expectation, it is at high elevation that we found the highest proportion of damaged seeds: seeds are more abundant at low elevation (see also Olejniczak et al., 2018), but the proportion of damaged/predated seeds is higher at high elevation. Bogdziewicz et al. (2019) working on *Quercus ilex* infested by a weevil across the entire Iberian Peninsula found a high seed predation at high elevation. Therefore, our finding of more predation where resources (seeds) are less abundant (see also Elzinga et al., 2005; Fagan et al., 2005; Kunin, 1999) matches the resource dilution effect (Otway et al., 2005) rather than the resource concentration hypothesis. That the weevils *Antliarhinus zamiae* do not cause more seed damage at low elevation where seeds are more abundant may be due to several factors linked to their biology and ecology. These factors may include: (i) how physiologically active the weevils are in understorey conditions versus in open habitats where solar energy is available, (ii) how effective the scents emitted by the seeds in attracting the weevils are, along an elevational gradient, (iii) how active the weevils are along an altitudinal gradient, etc. (Kareiva, 1983; Kunin, 1999) and finally, (iv) it could simply be that seed production is substantially higher at low elevation than at high elevation. In such scenario, the predation of a small number of seeds at high elevation would result in a high proportion of predated seeds.

Furthermore, we found evidence of more seed predation on plants with more leaves. We argue that this pattern may be linked to photosynthetic reactions in leaves. More leaves mean more carbohydrates synthetised which are then made available to other plant organs including seeds as resources or foods for the weevils predating these seeds. McArt et al. (2013) demonstrated experimentally a strong relationship between leaves and seed predation such that seed predation may be reduced by 77% when leaves are eaten by herbivores. They specifically demonstrated that the herbivory of leaves induces an accumulation of jasmonic acid and complex phenolics in reproductive tissues which make seeds less palatable to predators (McArt et al., 2013). These findings are in support of ours that more leaves correlate with more seed predation. There is evidence of some insects feeding on cycad leaves (mostly Lepidopteran larvae – both butterflies and moths; Bayliss et al., 2009; Donaldson & Bösenberg, 1995), and this folivory may trigger similar defence in cycad seeds. However, there is a need to conduct a similar study to that of McArt et al. (2013) to tell whether attacks of leaves of *E. villosus* by folivorous insects induce a defence mechanism in seeds such as the synthesis of chemicals that may increase seed unpalatability to predators.

We also found that habitat types correlate with seed pre‐dispersal predation such that more damaged seeds are found in close habitats. Several studies investigated habitat predisposition of plants to pre‐dispersal seed predations (e.g. Forget et al., 1999; Leimu et al., 2002). Leimu et al. (2002) reported higher seed predation of *Primula veris* by *Amblyptilia punctidactyla* in close habitats. *Amblyptilia punctidactyla*, the seed predator of *Primula veris*, is a generalist herbivore feeding on various other species including *Stachys, Aquilegia, Geranium* and *Erodium* species (Beirne, 1954), whereas the seed predator of *Encephalartos villosus* (*Antliarhinus zamiae*) is a specialist seed predator feeding exclusively on the *Encephalartos* gametophyte (Donaldson, 1992). Therefore, the fact that both plants (*P. veris* and *E. villosus*) show higher seed predation in close habitat suggests that the predisposition to seed predation may have to do with habitat types rather than the feeding regime of the seed predators (generalist vs. specialist).

Overall, seed predators may render up to 77% of seeds inapt for reproduction (Greig, 1993; McArt et al., 2013), thus potentially jeopardising the future of affected populations. This prompts the need for a continued commitment to understanding the predisposition of seeds to pre‐dispersal attacks by herbivores. We found that where seed production is high, the proportion of predated seeds is low, matching the pattern of resource dilution effect. Interestingly, an early study investigated the effects of seed predation on the population of two *Encephalartos* species, one savanna species – *E. cycadifolius* – and *E. villosus*, our model species in the present study (Raimondo & Donaldson, 2003). They found that the loss of seeds would have a minimal effect on the population dynamic of both *Encephalartos* spp. In contrast, the loss of adult individuals of both species would have a dire consequence on the populations of both species, a contrasting finding to what was reported for mangrove species in South Africa (Gaoue & Yessoufou, 2019) where harvesting adult mangroves is expected to have minimal effect on population dynamics most likely due to low reproductive values of adults. Therefore, adults of *Encephalartos* seem to have high reproductive values based on Raimondo and Donaldson's (2003) findings, but this needs to be tested in our study area as another step towards the generalisation of the demographic importance of adult cycads.

## AUTHOR CONTRIBUTIONS

**Kantakwa Grégoire Sadiki:** Data curation (equal); formal analysis (equal); investigation (equal); writing – original draft (equal). **Kowiyou Yessoufou:** Conceptualization (equal); formal analysis (equal); funding acquisition (equal); investigation (lead); methodology (lead); project administration (lead); resources (equal); software (lead); supervision (lead); validation (lead); visualization (lead); writing – review and editing (lead). **Terence N. Suinyuy:** Conceptualization (equal); funding acquisition (equal); project administration (equal); resources (equal); supervision (equal); writing – review and editing (equal).

## FUNDING INFORMATION

The National Research Foundation – South Africa, Grant #SRUG22051210107 to K.Y., and Grant UID 129403 to T.N.S.

## CONFLICT OF INTEREST STATEMENT

The authors declare that they have no conflict of interest.

## Supporting information


Tables S1–S4.

Data S1./

Data S2.

## Data Availability

Data analysed in the study and the R script used are available as Supplemental Information to this manuscript.
