# Supplementary material for: Resource dilution effect rather than resource concentration hypothesis explains the patterns of pre‐dispersal seed predation of an African cycad along an elevational gradient in South Africa
Source: Ecol Evol. 2024 Aug 27;14(8):e70209. doi: 10.1002/ece3.70209 (PMC11347868; doi:10.1002/ece3.70209)

**Table S1** Coefficients of the model of the number of leaves showing the effect size of each predictor


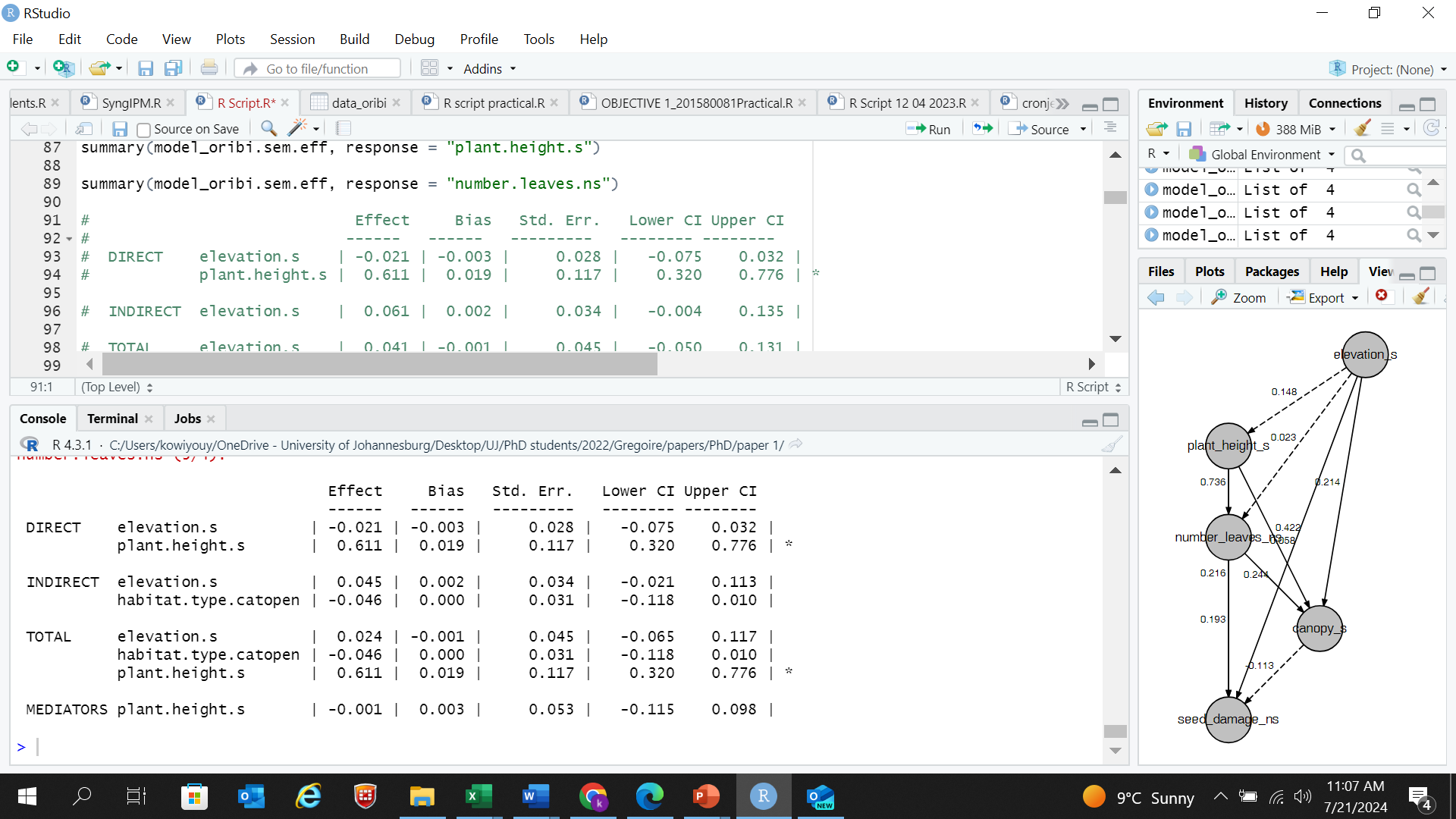


**Table S2.** Coefficients of the model of plant height showing the effect size of each predictor


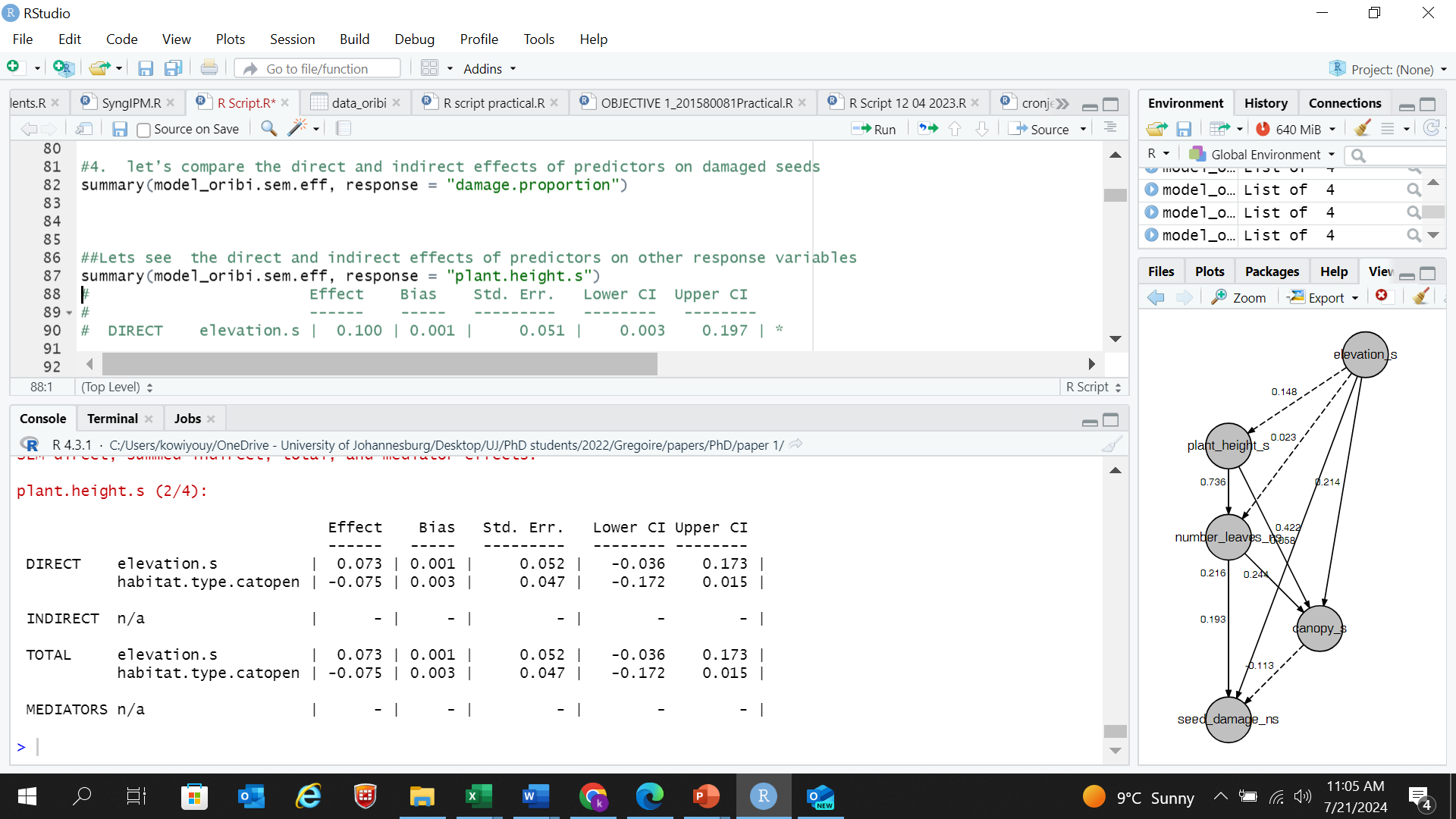


**Table S3.** Coefficients of the model of the plant canopy showing the effect size of each predictor


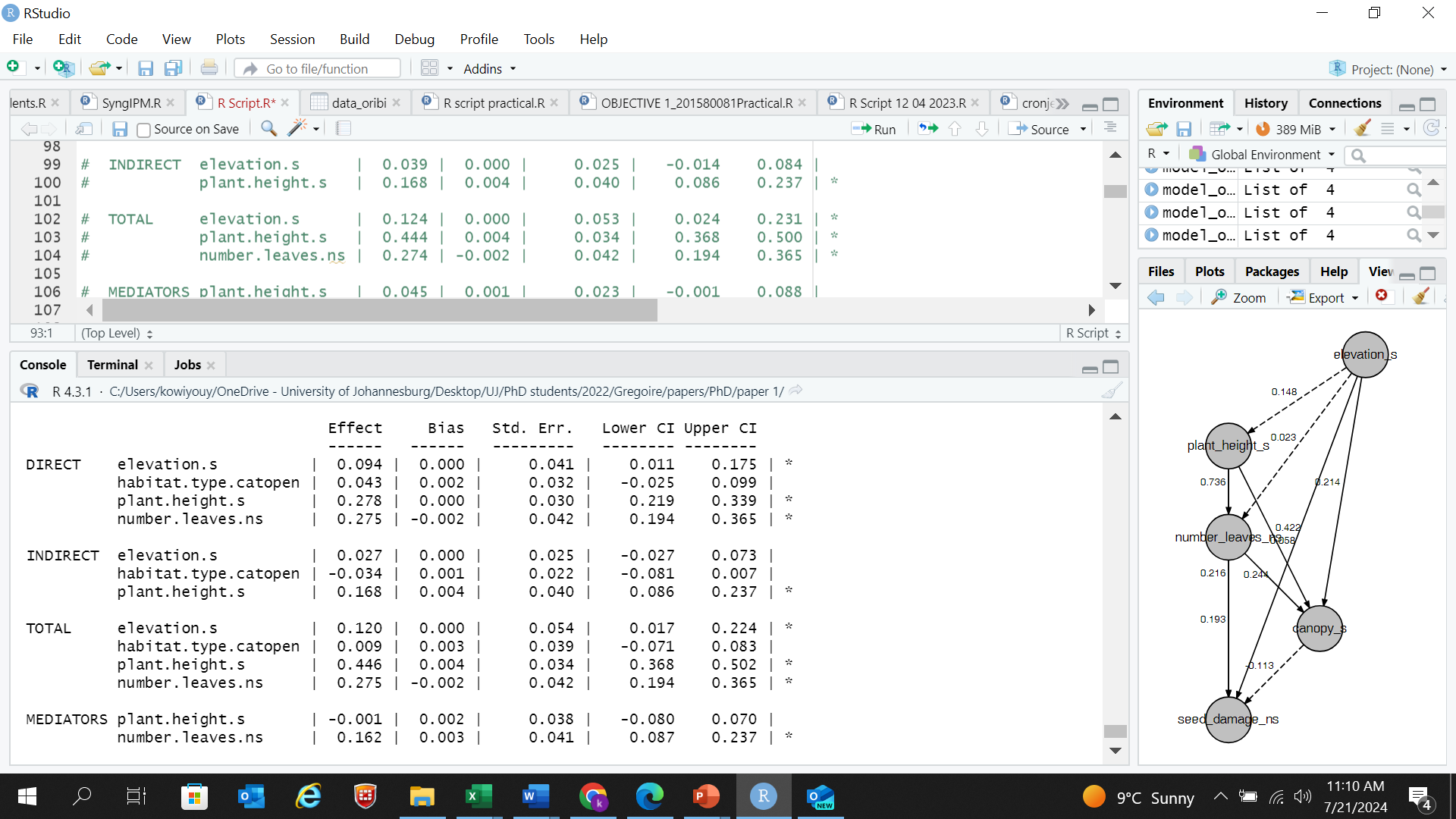


**Table S4.** Coefficients of the model of seed production showing the effect size of each predictor


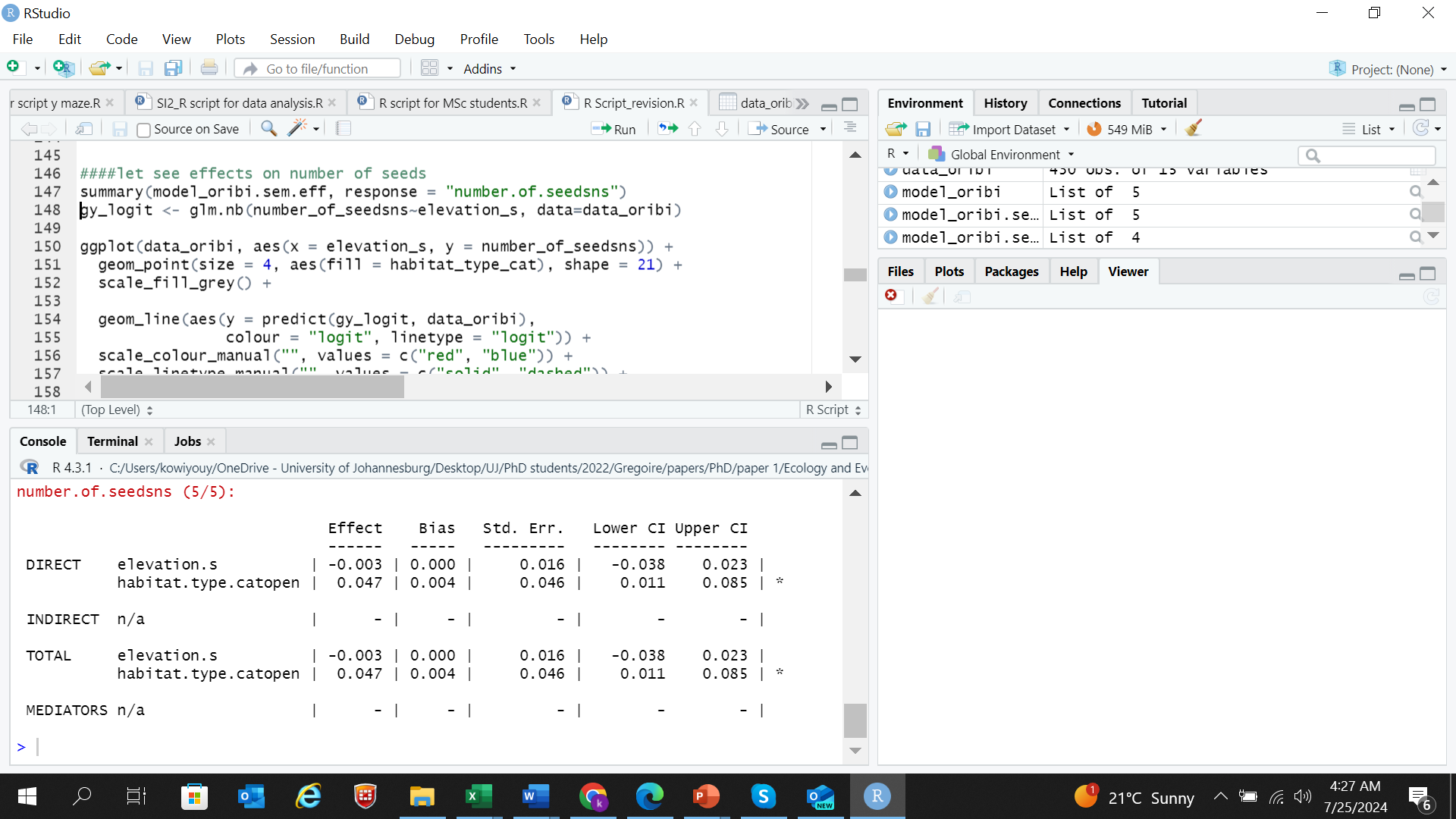

Supplement: Supplementary file 1 — Tables S1–S4. [file ECE3-14-e70209-s001.doc]
